# All-cause mortality risk in older patients with femoral neck fracture

**DOI:** 10.1186/s12891-022-05880-y

**Published:** 2022-10-28

**Authors:** Xin-ping Li, Ping Zhang, Shi-wen Zhu, Ming-hui Yang, Xin-bao Wu, Xie-yuan Jiang

**Affiliations:** 1 Department of Geriatrics, Beijing Jishuitan Hospital, 100035 Beijing, China; 2 Department of Orthopaedic and Traumatology, Beijing Jishuitan Hospital, 100035 Beijing, China

**Keywords:** Osteoporosis, Femoral neck fracture, Mortality

## Abstract

**Introduction:**

The mortality rate after hip fracture is high. However, the 1-year mortality rate after femoral intertrochanteric fracture and femoral neck fracture differs (Gibson-Smith D, Klop C, Elders PJ, Welsing PM, van Schoor N, Leufkens HG, et al., Osteoporos Int 25:2555-2563, 2014), although both are types of hip fracture. A previous real-world single-center prospective cohort study showed that older age and high Charlson comorbidity index score were risk factors for femoral intertrochanteric fracture. Additionally, therapy with zoledronic acid 5 mg (Aclasta) was a protective factor (Li XP, Zhang P, Zhu SW, Yang MH, Wu XB, Jiang XY, J Orthop Surg Res. 16:727, 2021). We wished to determine the risk factors for all-cause mortality in femoral neck fracture patients.

**Aim:**

To identify the risk factors for postoperative all-cause mortality in aged patients with femoral neck fracture.

**Materials and methods:**

We enrolled 307 aged patients with femoral neck fracture; 38 were lost to follow-up after 2–3 years. The patients’ general characteristics, bone mineral density, and anti-osteoporosis treatment after operation were recorded as potential risk factors. Kaplan–Meier curves and multivariate Cox proportional hazards models were constructed to analyze the influence of each factor on all-cause mortality.

**Results:**

This was a real-world single-center prospective cohort study showing that (1) most of the patients who died were male, older (mean age of the patients who died: 84.8 years vs. 77.9 years for survivors), and had more comorbidities compared with surviving patients. Previous fracture history, body mass index, femoral neck T score, hemoglobin and 25-hydroxy vitamin D levels did not differ significantly between patients who died vs. survived. (2) Differing from patients with intertrochanteric fractures, older patients with femoral neck fracture experienced no reduction in all-cause mortality with treatment with zoledronic acid.

**Conclusion:**

In Chinese patients with femoral neck fracture, physicians should pay careful attention to male patients, older patients, and those with high numbers of comorbidities.

## Background

Hip fracture is associated with significantly high all-cause mortality [1]; approximately 33% of men and 22% of women suffering a hip fracture will die within 1 year [2, 3]. Therefore, researchers have explored the risk factors for death to provide early interventions and decrease mortality. Hip fracture includes two main fracture types: femoral neck fracture and intertrochanteric fracture. Although often studied together, the two fracture types differ in anatomical location (femoral neck fracture is an intra-capsular fracture, and femoral intertrochanteric fracture is an extra-capsular fracture), surgical repair methods (femoral neck fractures are often treated with joint replacement or internal fixation, while intertrochanteric fractures are mainly treated with internal fixation), healing process (less callus after femoral neck fracture fixation and bone ingrowth in cementless femoral prosthesis and obvious callus in intertrochanteric fractures), and post-fracture mortality (lower mortality with femoral neck fractures compared with intertrochanteric fractures)[4]. Previously, our research team examined the risk factors for postoperative death in patients with intertrochanteric fractures, namely age, number of comorbidities, and receiving zoledronic acid (ZOL) after surgery, as anti-osteoporosis therapy. The purpose of this study was to explore the risk factors for all-cause mortality in patients with femoral neck fracture receiving the same treatment and during the same time range of admission.

## Patients and methods

### Study Population

A pelvic X-ray was obtained as soon as possible after admission to the emergency department. After a diagnosis of a femoral neck fracture, patients were admitted to the geriatric trauma unit, which involves co-management by geriatricians and orthopedic surgeons throughout hospitalization. No surgery was performed on the weekend. Fifty-five patients underwent cannulated screw internal fixation, and 219 patients underwent joint replacement.

All patients received osteoporosis health education. Seventy-one patients agreed to therapy with ZOL, 5 mg (Aclasta; Novartis Pharma, Basle, Switzerland), less than 3 days after surgery and supplementation with calcium and vitamin D, as the treatment group. The remaining 198 patients agreed to basic osteoporosis treatment comprising calcium and vitamin D supplementation and refused other anti-osteoporosis treatment.

### Inclusion and exclusion criteria

The inclusion criteria in this study were: (1) patients with acute, fragility femoral neck fractures (less than 3 weeks’ duration) and aged > 65 years; (2) unilateral hip fracture; and (3) patients taking no anti-osteoporosis therapies other than calcium and vitamin D supplementation.

The exclusion criteria were: (1) secondary osteoporosis; (2) high-energy fractures and/or multiple fractures; (3) pathological fracture caused by a malignant tumor; and (4) contraindications to anti-osteoporosis drugs.

### Follow-up Method and Endpoints

In this study, patients were followed-up in our outpatient service and by telephone. The primary endpoint was all-cause death, and the date of death or the date of the last telephone call determined the end of the follow-up. For the telephone follow-up, the patient’s name and operation time were recorded for identification, and the patient or their agent at the time of operation were contacted to provide information. The staff performing the follow-up remained unchanged throughout the follow-up.

### Potential confounders

Age, sex, body mass index (BMI), history of comorbidities (using the Charlson comorbidity index (CCI)), hip neck T score (dual-energy X-ray absorptiometry), fracture history, and 25-hydroxy vitamin D and hemoglobin levels were determined at admission. Use of ZOL and secondary fractures were recorded during the follow-up. Confounders were included in the final model if they changed the beta coefficient of the association by > 5%.

### Statistical analysis

Assuming a post-fracture all-cause mortality rate of 10% (underestimated on the basis of previous studies [3–5]), incidence of 45% (in accordance with the use rate of anti-osteoporosis drugs in the pilot study), hazard ratio of the risk factors of 0.3 (overestimated on the basis of a previous study [6]), two-sided α-value of 0.05, and a follow-up loss rate of 20% (overestimated to avoid too few cases), an estimated 275 cases were required to power 80% of the COX regression module of PASS 15.0 software (NCSS, LLC, Kaysville, UT, USA).

Cases with missing values were excluded from the analysis. The t-test was used to compare age, BMI, and CCI between the two groups. The *χ*^2^ test was used to compare gender, CCI ≥ 3, and the number of fractures before and after the current hip fracture between the two groups.

Kaplan–Meier survival curves were created first, then the statistically significant factors were analyzed using a multivariate Cox proportional hazards model. Forward and backward stepwise models were used to determine the most appropriate model. All statistical analyses were performed using SPSS version 19.0 (IBM Corp., Armonk, NY, USA) software, and p < 0.05 was considered statistically significant.

## Results

### General patient information

We enrolled 307 patients aged ≥ 65 years with femoral neck fracture in this study. All patients were admitted from May 2015 to December 2017. Thirty-eight patients were lost to follow-up, which lasted 22.3 ± 5.6 months; 21 patients could not be contacted owing to a change in their telephone number, and 17 patients did not answer or refused to answer the telephone. There were 187 women and 82 men with an average age of 78.4 ± 7.2 years; the oldest patient was 96 years old, and 13% of the patients had more than three comorbidities.

The patients who died were more likely to be male, older, and have a high number of comorbidities compared with survivors. A previous history of fragility fracture, BMI, total hip bone mineral density, hemoglobin and 25-hydroxy vitamin D levels, and therapy with ZOL did not differ significantly between the patients who died and the survivors (Table 1).

**Table 1:** General Condition of the femoral neck fracture patients

Status	Case M/F	Age years	Pre-fracture M/F		Re-fracture M/F		BMI kg/cm^2^	HipNeck-BMD g/cm^2^	HGB g/l	25OHD ng/ml	CCI ≥ 3 n(%)	Zoledronic acid(%)
Alive	250 (68/182)	77.9 ± 7.2		8/40	0/5	22.5 ± 3.4		-2.5 ± 0.8	125.6 ± 14.1	13.5 ± 7.3	26 (10)	65 (26)
Death	19 (14/5)	84.8 ± 4.2		3/2	0/0	21.5 ± 3.8		-2.6 ± 0.9	121.0 ± 15.1	11.3 ± 6.1	10 (53)	6 (32)
Total	269 (82/187)	78.4 ± 7.2		11/42	0/5	22.4 ± 3.4		-2.5 ± 0.8	125.2 ± 14.1	12.3 ± 7.3	36 (13)	71 (26)
p-value#	< 0.001	0.000		0.453	0.839	0.220		0.482	0.176	0.232	0.000	0.624

M, male; F, female; BMI, body mass index; BMD, bone mineral density; HGB, hemoglobin; CCI, Charlson comorbidity index; #?the compare between alive and death; continuous variables are mean (SD), categorical variables are numbers (percentage).

### All-cause mortality

Nineteen patients (14 men and 5 women) died during the follow-up period. The cumulative mortality rate was 7.1%. Of the 19 deaths, 4 deaths occurred within 3 months, 0 within 3–6 months, and 2 within 6–12 months. Acute cardiovascular events and pneumonia were the most common causes of death.

**Fig. 1:**
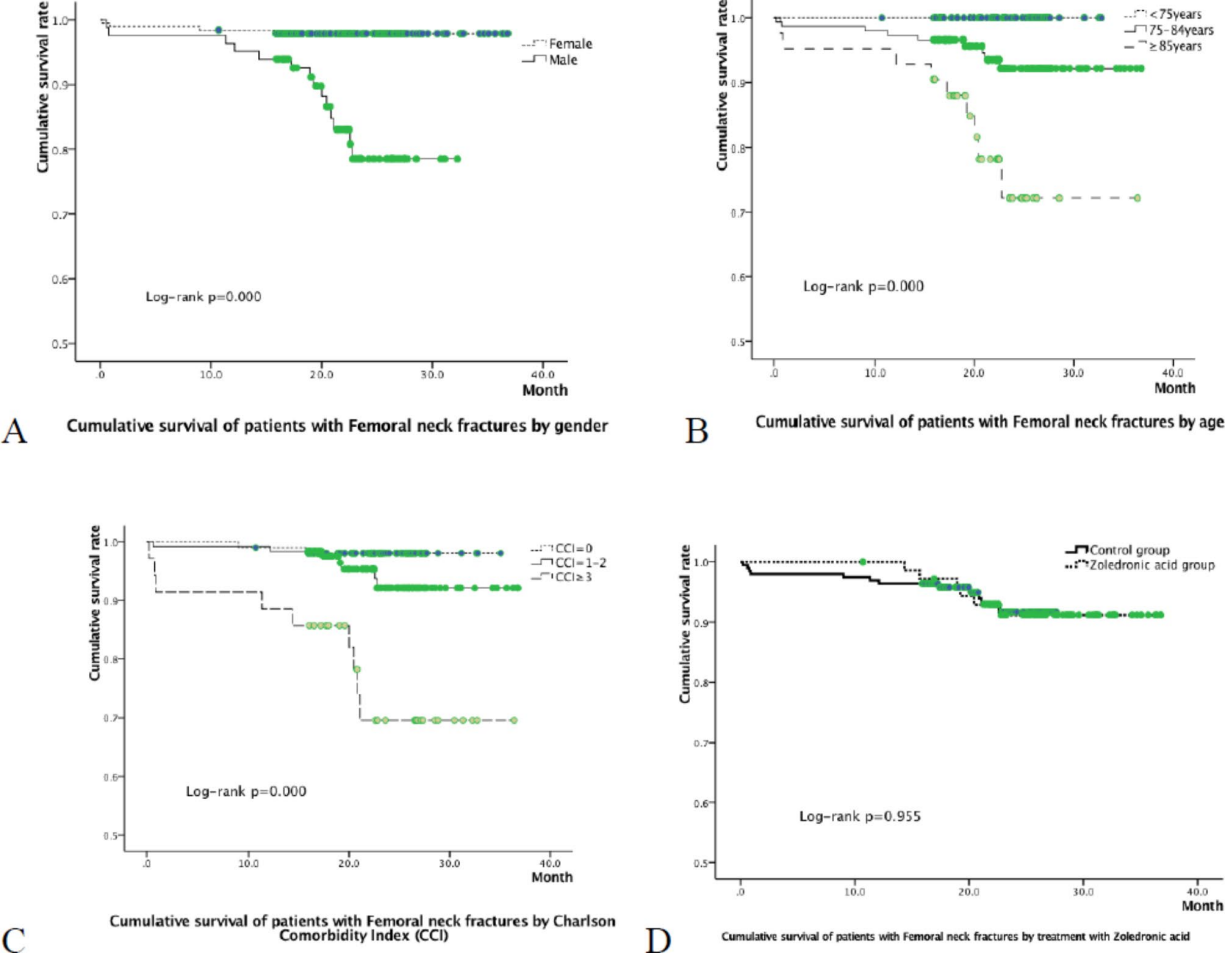
Cumulative survival of patients with Femoral neck fracture by gender (**A**), age (**B**), Charlson Comorbidity Index (**C**) and dosing Zoledronic acid or not (**D**)

Kaplan–Meier survival curve analysis showed that being male (P < 0.001; Fig. 1 A), older (P < 0.001; Fig. 1B), and having a CCI score ≥ 3 (P < 0.001; Fig. 1 C) were associated with a high mortality risk, whereas therapy with ZOL (P = 0.955; Fig. 1D) was not a risk factor for postoperative death.

Age, gender, CCI score, and therapy with ZOL were included in the multivariate Cox proportional hazards model. The results showed that gender (p = 0.001), age (p = 0.001), and CCI (p = 0.016) were associated with all-cause mortality; therapy with ZOL (p = 0.880) was not correlated (Table 2). Men had an 8.93-fold higher mortality risk than that for women; patients older than 85 years of age had a 4.21-fold higher mortality risk than that of younger patients; and patients with a CCI ≥ 3 had a 2.65-fold higher mortality risk compared with those with a CCI < 3.

**Table 2:** Multivariate Cox proportional Analysis of Risk Factors for Mortality in femoral neck fracture patients

Type	Variable	HR (95% CI)	P Value
Femoral Neck Fracture	Gender,male/female	8.93 (2.55–31.23)	0.001
	Age,≥85/75–85/<75years	4.21 (1.79–9.86)	0.001
	CCI,≥3/1–2/0	2.65 (1.20–5.88)	0.016
	ZOL,use/none		0.880

CI, confidence interval; HR, hazard ration; CCI, Charlson comorbidity index; ZOL ,Zoledronic acid; Blank cells indicate variables were not significant in the multivariate Cox proportional hazards models.

## Discussion

This was a real-world single-center prospective cohort study involving 307 patients; 38 patients were lost to follow-up after 2–3 years. The all-cause mortality rate after hip fracture surgery in this study was 7.1%, and the risk factors for death were older age, male sex, and high number of comorbidities.

Compared with most studies of all-cause mortality in patients with hip fracture, the mortality rate of the patients in this study was lower [1–3]. Even if all 17 patients who did not answer the follow-up call were considered as deaths, the all-cause mortality was still not high. We believe that orthogeriatric care [5, 7] and rapid operation models [8] are reasons for the low mortality rate in this study. We found that the risk factors for mortality and/or the proportions of specific risk factors changed under these models, such as the finding that pre-operative anemia was not a risk factor for all-cause mortality in our findings, and the Nottingham hip fracture score was adopted over time [9].

On the basis of our previous findings [10] and those in this study, age and the presence of comorbidities were risk factors for all-cause mortality in femoral neck fracture and intertrochanteric fracture; consistent with previous studies [11, 12]. These results suggest that more attention should be paid to older patients and patients with a high number of comorbidities.

Numerous studies [13, 14] have indicated that the mortality rate in men with hip fractures is higher than that of women; however, no explanation for this difference has been identified. Some studies showed that men had more comorbidities compared with women [15]. When there was no difference in patient age or the number of comorbidities between men and women, studies such as that by Endo et al. [16] showed that men with hip fractures were sicker compared with women, on the basis of the American Society of Anesthesiologists classification.

The Health Outcomes and Reduced Incidence with Zoledronic Acid Once Yearly – Pivotal Fracture Trial suggested that ZOL can reduce all-cause mortality in patients with hip fracture [6]. Our previous study of femoral intertrochanteric fracture confirmed this finding; however, this finding was not confirmed in the current study of patients with femoral neck fracture. The reasons for this difference are as follows: (1) ZOL mainly binds to hydroxyapatite crystals, especially at sites of active bone metabolism [17], and this drug plays a role in inhibiting the function of osteoclasts. However, femoral neck fracture is an intra-capsular fracture, and callus formation is significantly lower compared with femoral intertrochanteric fracture, which is an extra-capsular fracture. Therefore, the total amount of ZOL deposited in the bones of patients with femoral neck fracture is significantly lower compared with patients with intertrochanteric fractures, and the effectiveness of the drug’s intra- and extra-osseous effects is also lower. (2) The patients in this study received ZOL only once, and the survival curves (Fig. 1D) showed that the curves for the two fracture types were originally slightly separated and gradually overlapped after more than 1 year, suggesting that ZOL therapy should be continuous.

## Limitations

There are limitations in our study, as follows: (1) This was a real-world study, with no strict randomized grouping because it was not possible to perform a double-blind trial. This was because ZOL has a clear benefit for patients with osteoporosis, especially those with fragility fractures. (2) Many follow-up contacts were by telephone; therefore, there are subjective factors to consider. (3) As the family members of the patients who died were unwilling to speak with us, loss to follow-up led to underestimating the mortality rate and false negative results in the analysis of the risk factors for death.

## Conclusion

Femoral neck fracture and intertrochanteric fracture differ regarding the surgical methods for repair and the mode of healing. Physicians should pay special attention to male patients with femoral neck fractures who are over 85 years of age and who have a CCI ≥ 3. Improved compliance with anti-osteoporosis drug therapy is needed.

## Data Availability

The datasets analyzed during the current study are not publicly available but are available from the corresponding author on reasonable request.
